# SyS3DS: Systematic Sampling of Large-Scale LiDAR Point Clouds for Semantic Segmentation in Forestry Robotics

**DOI:** 10.3390/s24030823

**Published:** 2024-01-26

**Authors:** Habibu Mukhandi, Joao Filipe Ferreira, Paulo Peixoto

**Affiliations:** 1 Institute of Systems and Robotics, University of Coimbra, 3030-290 Coimbra, Portugal; 2 Computational Intelligence and Applications Research Group, Department of Computer Science, School of Science and Technology, Nottingham NG11 8NS, UK; 3 University of Coimbra, Department of Electrical and Computer Engineering, 3030-290 Coimbra, Portugal

**Keywords:** 3D LiDAR sensor, semantic segmentation, deep learning, LiDAR intensity

## Abstract

Recently, new semantic segmentation and object detection methods have been proposed for the direct processing of three-dimensional (3D) LiDAR sensor point clouds. LiDAR can produce highly accurate and detailed 3D maps of natural and man-made environments and is used for sensing in many contexts due to its ability to capture more information, its robustness to dynamic changes in the environment compared to an RGB camera, and its cost, which has decreased in recent years and which is an important factor for many application scenarios. The challenge with high-resolution 3D LiDAR sensors is that they can output large amounts of 3D data with up to a few million points per second, which is difficult to process in real time when applying complex algorithms and models for efficient semantic segmentation. Most existing approaches are either only suitable for relatively small point clouds or rely on computationally intensive sampling techniques to reduce their size. As a result, most of these methods do not work in real time in realistic field robotics application scenarios, making them unsuitable for practical applications. Systematic point selection is a possible solution to reduce the amount of data to be processed. Although our approach is memory and computationally efficient, it selects only a small subset of points, which may result in important features being missed. To address this problem, our proposed systematic sampling method called SyS3DS (Systematic Sampling for 3D Semantic Segmentation) incorporates a technique in which the local neighbours of each point are retained to preserve geometric details. SyS3DS is based on the graph colouring algorithm and ensures that the selected points are non-adjacent in order to obtain a subset of points that are representative of the 3D points in the scene. To take advantage of the ensemble learning method, we pass a different subset of nodes for each epoch. This leverages a new technique called auto-ensemble, where ensemble learning is proposed as a collection of different learning models instead of tuning different hyperparameters individually during training and validation. SyS3DS has been shown to process up to 1 million points in a single pass. It outperforms the state of the art in efficient semantic segmentation on large datasets such as Semantic3D. We also present a preliminary study on the validity of the performance of LiDAR-only data, i.e., intensity values from LiDAR sensors without RGB values for semi-autonomous robot perception.

## 1. Introduction

Wildfires have recently caused major natural disasters in countries such as the United States and in Mediterranean countries, such as Portugal, Spain, Italy, and Greece [1]. Over the past 25 years, there have been about 65,000 fires per year in Europe and about 18,000 fires per year in Portugal alone, with more than 100 victims since 2017 [2,3]. One of the measures to prevent wildfires is to encourage landscaping by actively reducing the accumulation of combustible material and identifying which plants and forest debris catch fire more easily than others. However, these and other adopted measures have not yet solved the problem as they lead to huge investments and focus heavily on human resources, and it is difficult to find employees willing to work in landscaping due to the harsh and dangerous conditions inherent to this job.

Semi-autonomous robots have become a promising solution in forestry, providing significant cost savings in the maintenance of forests [2,4,5]. They can also be particularly useful for performing tasks that it is difficult to find humans willing to do or that are dangerous for humans, such as tasks that pose a high risk of accidents and tasks that can lead to injuries, including back injuries, cuts, numbness, lacerations, and falls, without fully replacing humans. However, a semi-autonomous robot needs a perception system that allows it to navigate the forest and, for example, perform landscaping for fire prevention with the intention of actively reducing the accumulation of combustible material. An important module of a perception system is efficient semantic segmentation, as it enables the identification of objects of interest in the robot’s environment.

As average selling prices for LiDARs have declined and they are less affected by adverse weather conditions, they are increasingly being used for robotic perception, and they can capture more information than an RGB camera; therefore, they are increasingly being used for robot perception. A semi-autonomous vehicle with a 3D LiDAR sensor can detect whether a region is traversable or not to gain a better understanding of the real environment. Therefore, this work aims to address two problems: (a) the development of state-of-the-art LiDAR-based semantic segmentation techniques, which are crucial for tasks such as autonomous robot navigation and fuel detection in areas with complex terrain, and (b) the investigation of the performance of LiDAR-only data, i.e., intensity values from LiDAR sensors without RGB values, as a valid option for semi-autonomous robot perception. Semantic segmentation can also be used in other applications of field robots, e.g., in agriculture. Semantic segmentation in agriculture can enhance decision-making processes, may increase resource efficiency, and may contribute to sustainable and precise farming. Farmers can gain valuable insights to optimise crop production while minimising the environmental impact. In addition, efficient semantic segmentation can be used for intelligent real-time systems, such as augmented reality (AR). Efficient semantic segmentation in AR can improve scene understanding, object recognition, interaction, and the overall user experience. It may also enable AR systems to seamlessly integrate virtual and real elements, opening up new possibilities for innovative and practical applications.

**Figure 1:**
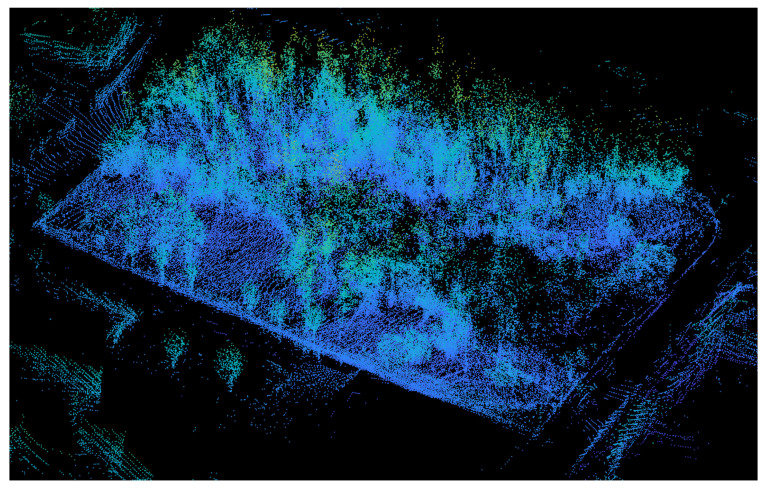
Typical example of 3D LiDAR data (i.e., point cloud) corresponding to a woodland environment. As can be seen, the 3D points are unstructured and sparse, and it is difficult to identify objects.

Deep convolutional networks have recently become state of the art in 2D structured computer vision tasks such as classification, object detection, and semantic segmentation. However, it is challenging to use them directly for the classification and semantic segmentation of unstructured point clouds [6]. Raw point cloud data are typically unstructured. These data are not arranged in a regular grid (unlike 2D images (Figure 1), where for every pixel there is a neighboring pixel on all four sides); their density is variable, i.e., objects closer to the sensor receive more points than those farther away; and they are unordered (i.e., permutation-invariant), which can be challenging for deep convolutional networks.

Another challenge is that almost all existing 3D point cloud semantic segmentation algorithms can process only small-scale 3D data (e.g., 4k points or 1 × 1 m blocks) and cannot scale to larger datasets (e.g., millions of points and up to 200 × 200 m) [7]. Therefore, it is only logical to sample these 3D points and select a subset of them. However, selecting a small subset of 3D points from a larger 3D point cloud may not capture the geometric structure of the point clouds and may miss relevant information that 3D LiDAR sensors capture.

There is also a lack of training and testing datasets that meet the following two requirements for semantic segmentation using 3D LiDAR data in forests: first, they should be acquired from outdoor woodland environments, and second, they should be annotated per point. For example, the TartanAir dataset [8] meets one of the two requirements; namely, it is annotated per point. However, many outdoor woodland environment data, including the QuintaReiFMD dataset [9], have outdoor woodland areas, but no annotations. To our knowledge, there are no known research reports on the use of 3D LiDAR data points acquired from outdoor woodland environments and no publicly available, labelled 3D LiDAR data for semantic segmentation tasks that meet both requirements. Furthermore, due to the complex and unstructured nature of outdoor woodland environments, labelling these data can be an arduous task that requires expertise.

To summarise, we propose a new point sampling technique called systematic sampling for 3D semantic segmentation (SyS3DS). A graph colouring algorithm is used in the sampling technique by building a k-d tree based on the difference between the x, y, and z coordinates. After creating the k-d tree, we compute the k-nearest neighbours for each point to preserve the local geometric features before using the sampled points as the input to a deep learning architecture.

## 2. Literature Review

Several strategies have been proposed in the literature to address the unique challenges presented by large 3D point clouds.

**Figure 2:**
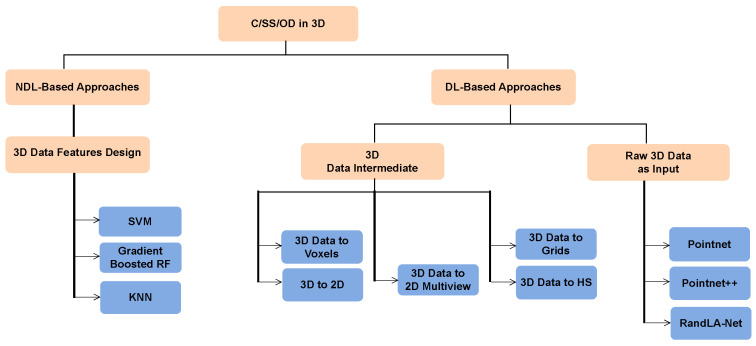
Taxonomy of classification, semantic segmentation, and object detection research with 3D LiDAR point clouds. C stands for classification, SS for semantic segmentation, OD for object detection, DL for deep learning, NDL for non-deep learning, SVM for support vector machines, RF for random forest, KNN for k-nearest neighbours, and HS for Hough space.

Figure 2 shows a taxonomy of the different approaches that exist in the literature to address this type of problem. We will be briefly reviewing these approaches in the following text; however, an in-depth discussion can be found in the survey paper by Bello et al. [10].

### 2.1. Non-Deep Learning-Based Approaches

Traditional machine learning algorithms, unlike deep learning methods that learn to select features on their own, require more human intervention to correctly learn how to select features. The basic idea in feature selection is to select those that maximise the discriminative power of each class. However, depending on the application, this can be difficult to achieve. For example, Aubry et al. [11] carefully defined features from a point cloud using the statistical properties of the points. However, it is not a trivial task to find optimal feature combinations that are specifically chosen to be invariant to certain transformations [12]. In the following list, we describe the two main approaches proposed in the literature for manual feature definition and selection for 3D data points and depth images as inputs to traditional machine learning algorithms:

- 3D key point detection: Lo and Siebert [13] use an improved version of the scale-invariant feature transform (SIFT) algorithm [14], namely 2.5D SIFT, to detect key points in a depth image. They created a discrete scale space representation of the depth image using Gaussian smoothing and the difference of Gaussian (DOG) method. The signal maxima and minima were detected within the DOG scale space. The key points were then validated and located by comparing the ratio of principal curvatures to a predefined threshold. The 2.5D SIFT algorithm achieved better fitting performance than the 2D SIFT algorithm [15]. Knopp et al. [16] first voxelised a mesh into a 3D voxel image. They then computed the second-order derivatives of each voxel using box filters with an increasing standard deviation, σ, and defined a saliency measure for each voxel and a scale σ based on the Hessian matrix. The local extrema were then determined and used to identify 3D SURF key points and their corresponding scales. Our own analysis has shown that both SIFT and SURF [17] for 3D data points have a computational complexity of $O(N^{3})$ for 3D data points, which is not suitable for point cloud preprocessing. After key point detection, the geometric information of the local surface around the key point can be extracted and encoded in a feature descriptor, which is another additional computational step;
- Novel statistical feature definition [18]: This approach is based on manual feature definition from 2D images. Features are defined by calculating the mean, median, standard deviation, coefficient of variance, skewness, covariance, kurtosis, correlation, and entropy of neighbouring 3D points. These calculations result in a set of feature vectors for a 3D point cloud. This set of features is then input to a feature optimisation step before being passed to a machine learning model. Like the other manual feature definition approaches, this one is computationally intensive and dependent on human supervision.

The best-known algorithms for non-deep learning are the gradient-boosted random forest algorithm [19,20], support vector machines [21] and k-nearest neighbours [22]. In addition to manual feature definition, a post-processing step, such as clustering [23], is required to improve the classification results of traditional machine learning methods, which also increases their computational costs.

### 2.2. Deep Learning-Based Approaches

Traditional machine learning algorithms are not well-suited for large amounts of data because they perform simple, mostly linear inference. In particular, for 3D point clouds, it can be difficult to learn complex features [24]. Deep convolutional networks have recently been shown to be effective when applied to structured 2D computer vision data. Therefore, researchers have turned to deep learning to process 3D point data [25]. The research can be mainly divided into two categories: methods that require the 3D data points to be converted into a more compact intermediate representation before being input into a deep learning model and methods where the raw 3D points are directly input into a deep learning model.

#### 2.2.1. Converting 3D Data into an Intermediate Representation

One of the two categories of approaches to input 3D point data into a deep learning model involves converting the 3D data points into a more compact intermediate representation before inputting them into a deep learning model. Some of the proposed approaches are described in this section.

##### 3D Data as 2D Images or Voxels

This method proposes the projection of 3D data points onto 2D images or 3D voxels to avoid irregularities in the 3D data. Moreover, processing 3D data points as images is computationally efficient because much research is being done on 2D data in the field of computer vision and deep learning using convolutional neural networks. Although this method has produced impressive results over the years, it makes the data unnecessarily voluminous, and much of the useful information contained in the 3D data points is lost. In addition, converting 3D data points into 2D images, as described by Li et al. [26], and into 3D voxels, as described by Graham et al. [27], leads to poor results because the 3D data are too sparse. Furthermore, these approaches are computationally intensive and may not be well suited for real-time systems [6].

##### 3D Data as 2D Images from Multiple Views

The multiview convolutional neural network (MVCNN) method developed by Qi et al. [28] is reported to provide better performance for object detection tasks than methods that represent 3D data as voxels or images. The idea is to represent a 3D data object as images obtained from multiple viewpoints. The authors used 12 viewpoints of an object and trained convolutional neural networks (CNNs) on the data as images. Each of the 12 viewpoints was used as input to an independent CNN, and the outputs of these 12 CNNs were then used as input to another CNN for segmentation. It is nontrivial to extend this method to 3D tasks such as point classification and shape completion.

##### 3D Data as Grids

Ben-Shabat et al. [29], who proposed 3DmFV (three-dimensional points as Fisher vectors), have found that converting 3D data to a grid does not have to involve working directly with raw 3D data as input to a deep learning model; nor does the data need to be projected onto 2D images. Also, this method has the advantage of being reversible to restore the original raw 3D data points, unlike the method of rasterising 3D data onto images. 3DmFV transforms 3D data to a grid using Fisher vectors and can revert from this grid to the 3D data points. This method performs similarly to the methods that project 3D data to 2D images or voxels. However, it cannot work in real time.

##### 3D Data to Hough Space

Other researchers working on the classification of 3D objects from point clouds, such as Song et al. [12], have proposed transforming LiDAR data points into the Hough space. First, they project the 3D data onto the x–z plane. Second, they map the x–z plane onto the Hough space. Finally, they use the accumulator count of the individual curves in the Hough space as the input to a deep learning model. However, this method cannot distinguish between trees and walls because these objects produce similar outcomes in the Hough space.

#### 2.2.2. Raw 3D Data as Input

Recent research suggests that it is possible to use 3D data points directly as input to a deep neural network. The problem of data irregularity has been studied and solved by modelling a symmetric function and designing a transformer network. The authors of the PointNet [6] paper, which is a pioneer work in the field of using raw 3D data points as input to artificial neural networks (ANNs), have developed a promising approach that directly processes raw 3D point clouds. The authors proposed using a function that models a symmetric function using shared multilayer perceptrons (MLPs). A symmetric function is used to account for the permutation invariance of 3D points, and a spatial transformer network is used to correct for affine transformations that may occur in 3D data points. The PointNet approach, while computationally efficient, does not capture the geometric context for each point because the symmetric function only captures the maximum features and discards the fine local features [7,30]. In Pointnet++ [30], which is an extension of Pointnet, each local region is passed independently to a symmetric function. Both Pointnet and Pointnet++ suffer from the problem that they can only be trained and work on small-scale point clouds.

To overcome the challenge that the geometric context is not captured for each point, many subsequent ANNs have been introduced [30,31,32,33,34]. They all achieve promising results in semantic segmentation, but they are limited to small 3D point clouds (e.g., processing times range from 10 to 200 s for these ANNs to process $10^{6}$ points on an NVIDIA RTX2080Ti) and cannot be easily scaled to larger point clouds [7], which makes them not feasible for use in an UAV or semi-autonomous robot applications. The main reason for this is that almost all of them use computationally intensive or memory-inefficient sampling techniques to select points and add them to the subset of points used as input to the deep ANNs. The following are some of the methods proposed in the literature for sampling 3D data points:

- Farthest point sampling (FPS): To select a subset of K points from the N original points, the algorithm returns a reordering of points $p_{1},\ldots ,p_{k},\ldots ,p_{K}$ such that each $p_{k}$ is the farthest point from the k−1 points already selected. FPS has been widely used in the literature, including in Pointnet++ [30] and Pointcnn [25] for semantic segmentation tasks. Despite the good coverage of an entire point cloud and the good representation of the data, the computational complexity is $O(N^{2})$. The authors of RandLA-Net reported that, for a large point cloud ($N\approx 10^{6}$), this sampling algorithm takes up to 200 s to process on a single NVIDIA RTX2080Ti GPU. Therefore, it cannot be used to sample large point clouds in real time;
- Policy gradient-based sampling: To select a subset, this method formulates the sampling operation as a Markov decision process. It sequentially learns a probability distribution to sample the points. The RandLA-Net authors report that for a sample of 10% of $10^{6}$ points, the exploration space is 105106, which is difficult for a neural network to converge to;
- Inverse importance sampling: To select a subset of *K* points from all *N* points, this algorithm orders all *N* points by their distance to a given reference point. The distance of each point becomes its density. All points are reordered according to their density. Then, the top *K* points are selected [35]. The computational complexity is on the order of O(N). The authors of RandLA-Net found through experiments that this algorithm takes 10 s to process $10^{6}$ points. Compared to farthest point sampling and policy gradient-based sampling, this algorithm is computationally efficient. However, it is sensitive to outliers and also not suitable for use in a real-time system;
- Random sampling: This method randomly selects K points from N original points. The method runs in O(1), constant time, and is independent of the number of points, so it can be scaled to any number of points. Compared to other sampling algorithms in the literature, it is the most computationally efficient method. According to the authors of RandLA-Net, it takes only 0.004 s to process $10^{6}$ points.

Landrieu et al. [36], who introduced large-scale point cloud semantic segmentation with superpoint graphs (SPG), proposed a technique to deal with large-scale raw 3D points using superpoints, which are similar to superpixels in 2D images, as inputs to a deep convolutional neural network. The creation of superpoints is very computationally intensive due to a pre-processing step that partitions the points by geometric positions [7]. Moreover, it can be difficult to detect a whiteboard on a white wall using superpoint partitioning [30].

The authors of RandLA-Net, which uses the random selection of raw 3D points to be able to run in real time, have found a solution to this problem that has been shown to work better than previous methods. The authors used a random selection of points and dropped the unselected points before inputting them into a deep neural network. This method has better accuracy than the best-performing state-of-the-art methods for the semantic segmentation of 3D data points, including the rasterisation of 3D data in image methods using only 3D point data as input.

In our work, we input raw 3D data into our deep learning network. As mentioned in the introduction (see Section 1), we use a systematic method of sampling points that can scale and process even larger amounts of 3D data in real time for efficient semantic segmentation.

### 2.3. Dataset

As we saw in Section 1, there is a lack of 3D datasets of outdoor woodland environments annotated per point. Therefore, we will first use known datasets from the autonomous driving research community to test our proposed method and compare it to existing 3D deep learning algorithms. Furthermore, self-driving car datasets contain objects such as vegetation, trees, people, and roads that may also be present in outdoor woodland environments.

We plan to evaluate our proposed work against a large public dataset, Semantic3D [37], to be fair in comparison to RandLA-Net. The Semantic3D dataset consists of 15 point clouds for training and 15 for online testing. Each point cloud contains up to 100 million points covering up to 160 × 240 × 30 m in real 3D space. The raw 3D points belong to 8 classes and contain 3D coordinates and RGB information and intensity. For simplicity, the authors of the state-of-the-art method used only the 3D coordinates and colour information to train and test their networks. The mean intersection over union (mIoU) and overall accuracy (OA) of all classes are used as standard metrics. Table 1 describes the classes of the Semantic3D dataset.

### 2.4. Comparative Evaluation of State-of-the-Art Approaches

Some of the works in the literature on 3D data are summarised in Table 2, including summaries of the performance of deep learning-based strategies on the SemanticKITTI dataset; Table 3 summarises the performance of different deep learning-based strategies on the Modelnet40 dataset [38]. Table 4 presents a summary of the different deep learning based solutions in the literature. It presents a comparative analysis of the design features of deep learning-based strategies.

There are research works, such as those on FG-Net [40], Qiu et al., 2021 [41], and KPConv [42], that show mIOU values of more than 72.23 on Semantic3D. However, FG-Net uses inverse density importance sampling (IDIS). The authors of RandLA-Net have found in experiments that the IDIS algorithm takes 10 s to process $10^{6}$ points. Although IDIS is computationally efficient compared to farthest point sampling and policy gradient-based sampling, the algorithm is sensitive to outliers. Qiu et al., 2021 [41], used farthest point sampling (FPS), which does not run in real time. The authors stated that they wanted to optimise this model’s efficiency for real-time applications in the future. The authors of KPConv do not seem to provide any information on its runtime performance for large datasets, such as Semantic3D, and seem to have used a block partition.

## 3. Systematic Selection of 3D Points

As mentioned earlier, it is difficult to process larger amounts of 3D data points and make inference in real time. Therefore, we propose a 3D point selection approach to select a subset of points, which is a better method for point selection than the random point selection proposed in RandLA-Net and has the advantage of being able to scale to larger point clouds by saving training time and significantly reducing the number of parameters. We also ensure that the order of input of 3D data points does not affect the performance of the segmentation algorithm since the geometric features of the data points are preserved.

Our proposed sampling algorithm is based on the graph colouring algorithm [43] to perform point selection, since we want to ensure that we obtain a subset of points that are representative of the 3D points in the scene. This algorithm ensures that the points we obtain are part of the skeleton of every object in the scene, which PointNet calls critical points. According to PointNet, only part of the skeletons of objects is sufficient to detect objects in the scene, since the critical points are not affected by density variations in the point cloud.

In addition, it has been shown that not all points are necessary for the correct detection of an object. PointNet has shown that up to 40% of points can be omitted or occluded without affecting performance and that up to 60% can be omitted without significantly reducing object detection performance. These results were obtained without considering the encoding of local geometric features, as we propose in our approach.

A graph is a nonlinear data structure with at least one node. In a graph, there may be nodes that are connected to each other; this connection is called an edge, E. A node is sometimes called a vertex, V. Formally, a graph consists of a set of vertices and edges. A graph is denoted as G(E,V). Two nodes are considered adjacent if they have an edge between them. The adjacent nodes are also called neighbours. The number of edges a node has is called the degree. A tree is a special type of graph that has no cyclic connections between nodes. A k-dimensional tree, or k-d tree [44], is a binary tree of points with more than one dimension. Since 3D LiDAR points are three-dimensional data, a k-d tree is a very effective way to construct a 3D data point graph using the nearest neighbour algorithm.

**Figure 3:**
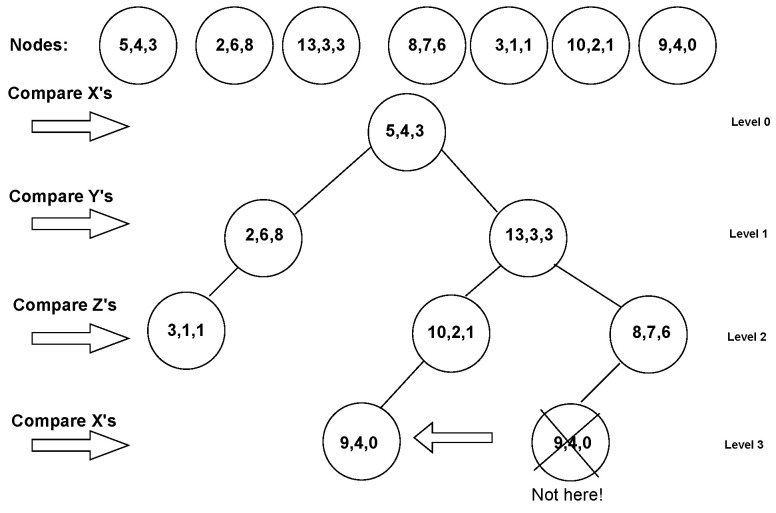
A k-d tree from three-dimensional data. When inserting the 3D points, the first node becomes the root node at level 0, and the next node goes to the left if its x-coordinate is smaller than the x-coordinate of the root; it goes to the right if its x is larger than the x of the root. At level 1, a node’s y-coordinate is compared with the y-coordinates of the node at the level. If they are smaller, we go to the left, and if they are larger, we go to the right. On level 2, the z-coordinates are compared. On level 3 we find the x coordinates that are compared as on level 0. Thus, the process repeats. Note that this algorithm makes a precedent of placing a node closer to its nearest neighbour throughout the process described above, as can be seen with nodes 9, 4, and 0.

The process of constructing a k-d tree from three-dimensional data has a complexity of O(NlogN), as can be seen in Figure 3, where N is the number of nodes. After creating a graph, we used k-nearest neighbours to find the nearest neighbours for each point (to save calculations, we used the square distance instead of the square root to find the Euclidean distance). RandLa-Net randomly selects $10^{5}$ points for each point cloud; therefore, we chose varying numbers of neighbours (i.e., from k = 14 to k = 81 depending on the total number of points in the point cloud) for each point cloud to sample ≈100 k points. The systematically sampled points were then used as inputs to a shared MLP. Our algorithm ensures that the selected points are non-adjacent, which can be essentially compared to the FPS algorithm, with the difference that our method is faster. To take advantage of the ensemble learning method proposed by Yang et al. [45], we passed a different subset of nodes for each epoch. This leverages a new technique called auto-ensemble, where ensemble learning is created as a collection of different learning models in one rather than tuning different hyperparameters individually during training and validation.

## 4. Local and Global Information Aggregation

In addition to systematically selecting points, we preserved local geometric structures using local spatial encoding (LocSE) and attentive pooling to automatically preserve useful local features. We also stacked multiple LocSE units and attentive pooling into an extended residual block, significantly increasing the effective receptive field for each point. Each point sampled in the systematic selection of points is considered a reference point. We refer to each reference point as point $p_{i}$ and each of its neighbours as $p_{i}^{k}$. In our work, the step of finding the nearest neighbours does not take any additional computation time since the nearest neighbours are already computed during the systematic sampling, unlike RandLA-Net, which performs the computations in this step.

**Figure 4:**
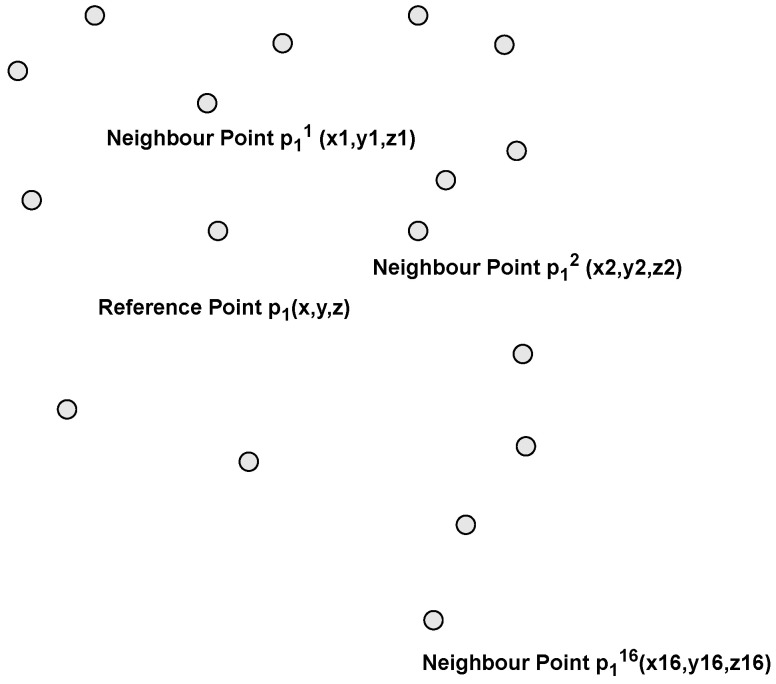
A reference point with its nearest neighbours. The neighbours’ x, y, and z positions are encoded as shown in Equation (1).

In order for the deep neural network to learn the relative position of each point and its geometric features, we need to preserve the sampled points and their considered neighbours because during the selection of a subset of points, many points are discarded. To obtain the relative positions, the x, y, and z positions of the 16 neighbours are concatenated as features in the feature map, along with the x, y, and z positions of the reference point as it can be seen in Figure 4. After we performed a heuristic hyperparameter search, we found that 16 neighbours is the number of neighbours that provides the best results. In addition, the Euclidean and Manhattan distances of a selected point relative to each of the reference point’s nearest neighbours are encoded into the feature to complete the feature map.

**Figure 5:**
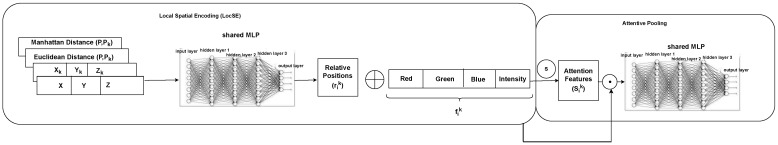
Attentive pooling to produce attentive features instead of using maxpooling. ⊕ is the concatenation operation, ⊙ is the dot product operation, and ⓢ is the Softmax layer.

Relative point position encoding:

(1) $$r_{i}^{k}=\mathrm{MLP}(p_{i}⊕p_{i}^{k}⊕\left(p_{i}-p_{i}^{k}\right)⊕∥p_{i}-p_{i}^{k}∥)$$

where ⊕ is the concatenation operation, ∥.∥ is the Euclidean distance, $\left(p_{i}-p_{i}^{k}\right)$ is the difference between the centre and the neighbouring point k, $p_{i}$ is the selected point, and $p_{i}^{k}$ is each of the k = 16 neighbours. MLP() is the input for the multilayer perceptron, and $r_{i}^{k}$ is the encoding of the relative point position. This encoding is concatenated with the other features, such as the RGB colours of each point and their intensity values, as shown in Figure 5.

The following equation introduces the concatenation process:

(2) $${\hat{f}}_{i}^{k}=r_{i}^{k}⊕f_{i}^{k}$$

where $f_{i}^{k}$ represents the RGB value and intensity value for each point and ${\hat{f}}_{i}^{k}$ is the point feature augmentation.

$S_{i}^{k}$ is the attention score from the Softmax outputs of a shared MLP, given by

(3) $$S_{i}^{k}=g\left({\hat{f}}_{i}^{k},w\right)$$

where *g* is the function estimated from a shared MLP with the input features ${\hat{f}}_{i}^{k}$, and *w* represents the learned weights after training the shared MLP.

Instead of selecting only the maximum features and ignoring the rest, we give each feature ${\hat{f}}_{i}^{k}$ a weight according to its importance by concatenating the attention scores of each feature with its corresponding feature (see Figure 5).

## 5. Deep Learning Architecture

Instead of max-pooling to approximate the underlying symmetric function in the data, we used attentive pooling, inspired by Engelmann et al. [46]. The reason for this is that max-pooling tends to drop the features that did not respond the most. Therefore, it becomes difficult to integrate the neighbouring features, which results in losing much of the information. Since we only used a subset of points, we needed all the neighbouring information we collected. This technique is inspired by the work of Yang et al. [47]. Attentive pooling uses an attention mechanism to automatically learn important local features.

**Figure 6:**
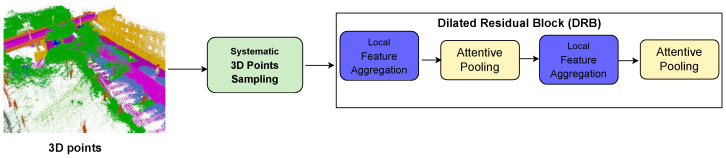
Dilated residual block containing a module for local feature aggregation and attentive pooling repeated twice to increase geometric feature encoding. To avoid overfitting, we avoid repeating this more than twice. Green boxes represent proprietary work, blue boxes represent work adapted from Yang et al., and yellow boxes represent work adapted from Engelmann et al.

We needed to encode the geometric information of many more neighbours, as we selected only a subset of points from an entire scene. The dilated residual block is a repeat of the local feature aggregation module and attentive pooling (see Figure 6). The subsequent local feature aggregation module receives input from the output of the previous attentive pooling. Therefore, the number of neighbours collected as features after repetition increases from *n* neighbours to $n^{2}$ neighbours.

**Figure 7:**
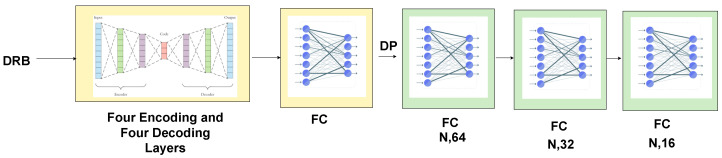
Encoder and decoder for downsampling and upsampling the features of the input points for the semantic segmentation task. (N, D) stands for the number of points and the feature dimension, respectively. FC stands for fully connected layer. DP stands for dropout layer. DRB stands for dilated residual block. N is the number of 3D points. Green boxes represent proprietary work, and yellow boxes represent work adapted from RandLA-Net.

We used four encoding layers and four decoding layers. Selected subsets of points and the dilated residual block were input to each encoding layer. In the encoding layers, the filter size (feature map) was gradually increased from 8 to 32 to 128 to 256 to 512, and the number of points was downsampled using strided convolution from N to N/4 to N/16 to N/256. The four decoding layers were the mirror image of the encoding layers (see Figure 7). The number of points was gradually upsampled from N/256 to N/16 to N/8 and back to N to perform semantic segmentation. The output of the last decoding layers was connected to a fully connected layer, which was connected to three other fully connected layers that preceded each other to predict the class for each point. This combination of decoder/encoder and four fully connected layers produced the best performance on the Semantic3D dataset after an extensive search for hyperparameters.

## 6. Experimental Evaluations

In Table 5, we summarise the evaluation of the computational complexity of our network on real, large-scale 3D point clouds for semantic segmentation. Specifically, we evaluated our network on the SemanticKITTI dataset [48], taking as a reference the complexity reported by RandLA-Net for a fair comparison. We only compared our work with RandLA-Net because the other deep learning architectures proposed in the literature used computationally intensive sampling techniques, such as farthest sampling algorithms, and are therefore not suitable for large sets of 3D points, as described in the Literature Review section. In contrast, RandLA-Net uses a random sampling algorithm that is suitable for large sets of 3D points. The SemanticKITTI dataset includes 43,552 scans divided into 21 sequences. Each scan has about 100,000 points distributed over a volume of 160 by 160 by 20 m. The sequences are divided into a training set, containing sequences 00 to 07, 09 and 10 (19,130 scans), a validation set containing sequence 08 (4071 scans), and sequences 11 to 21 for testing (20,351 scans). The dataset includes 19 classes, including cars, road signs, vegetation, people, terrain, cyclists, roads, sidewalks, etc. Our algorithm has higher computational complexity than inverse density importance sampling and random selection. However, inverse density importance sampling is sensitive to outliers, and random selection is not a systematic approach to point selection. Our algorithm is not sensitive to outliers, has reasonable complexity, and works similarly to random selection in real time.

We used a GeForce RTX 3090 Nvidia GPU with 24 GB of memory for both training and testing the Semantic3D dataset. We conducted four experiments. In the first experiment, we used the 3D coordinates and the colour information of each point to train and test our network, similarly to other researchers. The mean intersection over union (mIoU) and overall accuracy (OA) of all classes were used as standard metrics.

In Table 6, we summarise the results of the mIOU of our network on real, large-scale 3D point clouds for semantic segmentation. Specifically, we evaluated our network on the Semantic3D dataset [37] against the mIOU and IOU for each individual class reported by RandLA-Net for a fair comparison. It can be seen that our network outperforms RandLA-Net, which represents the state of the art in all eight classes. Further improvements are needed for the low vegetation class. Like RandLA-Net, our network performs poorly because of the limited data available for training in this class.

For the second experiment, only the 3D coordinates of each point were used to train and test our network since most 3D datasets do not contain colour information. The mean intersection over union (mIoU), IOU for each individual class, and overall accuracy (OA) of all classes were used again as standard metrics.

In Table 7, we summarise the mIOU results. The mIOU decreased by about ten percentage points to 62.25, as did the accuracy of each individual class.

For the third experiment, the 3D coordinates of each point and the intensity values were used to train and test our network as LiDAR-only data. The mean intersection over union (mIoU), IOU for each individual class, and overall accuracy (OA) of all classes were used once again as standard metrics.

In Table 8, we summarise the mIOU results. The mIOU decreased only about three percentage points to 68.15 compared to 72.23 for 3D coordinates with colour information. This shows that intensity values can perform almost as well as 3D coordinates with colour information even without colour information.

For the fourth experiment, we used input fusion with 3D coordinates, colour information, and intensity values to train and test our network. The mean intersection over union (mIoU), IOU for each individual class, and overall accuracy (OA) of all classes were used here as well as standard metrics.

In Table 9, we summarise the mIOU results. The mIOU here is lower than that of 3D coordinates with colour information and that of 3D coordinates with intensity values. Notably, our network also achieves superior performance on six of the eight classes, except the man-made and natural classes.

Table 10 summarises the benchmarking of our network with the state-of-the-art RandLA-Net on the Semantic3D (reduced-8) dataset with RBG features as the baseline for the four experiments we performed. Our first experiment provides the best mIOU, the best overall accuracy, and the best IOU of the man-made class, natural class, high vegetation class, low vegetation class, scanning artefacts class, and cars class. The second experiment provides the best IOU for the hardscape class. The fourth experiment provides the best IOU for the buildings class. It can also be observed that our network without RGB or intensity performs better than our first experiment for objects with classes with blocks, such as buildings and hardscape.

**Figure 8:**
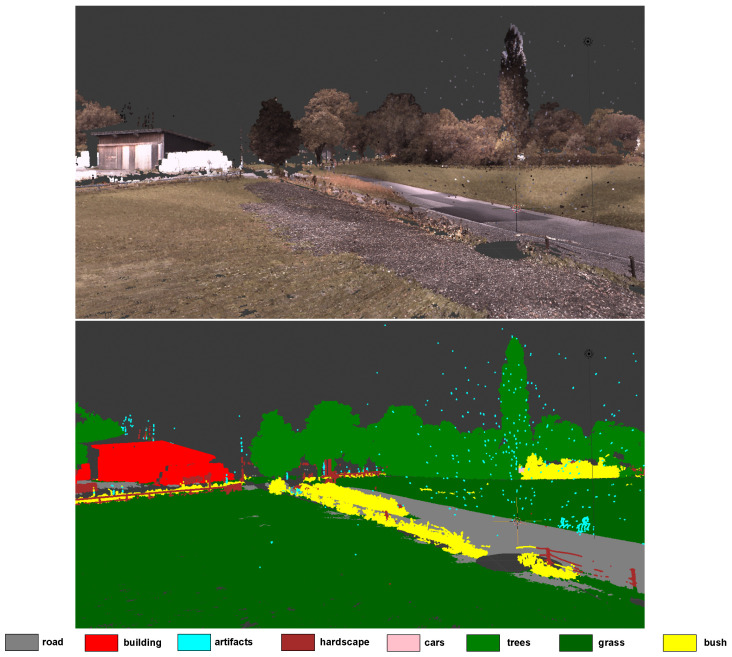
Qualitative analysis of our network from one view for a scene showing bushes, buildings, trees, grasses, hardscape, artefacts, and roads.

**Figure 9:**
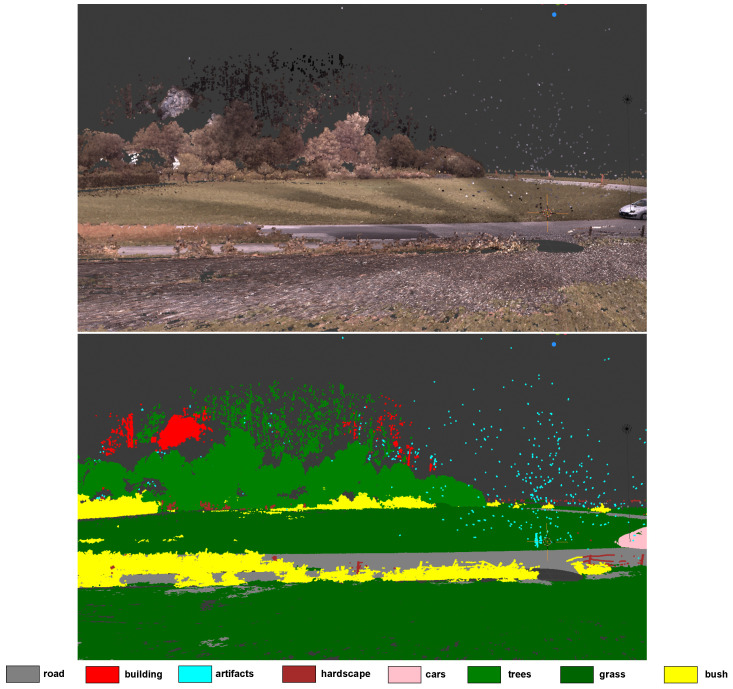
Qualitative analysis of our network from another view in the scene showing bushes, trees, grasses, artefacts, roads, and a car. It can also be seen that a rock on a hill is being classified as a building since the rock resembles some buildings in the dataset.

Figure 8 and Figure 9 enable a qualitative evaluation of the performance of our network on the test set of the Semantic3D dataset for the first experiment (XYZ + RGB). In general, our network seems to produce good quality results but can mistake things like hills as buildings. This could be due to the RGB channel features in the Semantic3D dataset that make hills look like old buildings. In some rare cases, our network may also mistake bushes for grass.

The qualitative analysis can also been viewed in the video, see link in Appendix A.

Table 11 summarises the runtime of SyS3DS on a GeForce RTX 3090 Nvidia GPU with 24 GB memory when tested on the Semantic3D (reduced-8) dataset with RBG features. It can be seen that 24 frames per second can be processed during inference.

## 7. Conclusions and Future Work

In this paper, we have shown that the systematic sampling of 3D data points outperfoms a simple random selection of points and has the advantage that large point clouds can be used as input and processed in real time. Our findings strongly support the key tasks of navigation, situational perception, such as the identification of flammable materials, the coordination of robotic teams, and decision-making by providing fine-grained information about the local environment. It also provides efficient semantic segmentation that can be used to provide decision-making modules with task-oriented context to inform the actions that the robot should enact.

The baseline of the state of the art for efficient semantic segmentation shows that further improvements are needed for the low vegetation class. Similarly to RandLA-Net, our network performs poorly because of the limited data available for training this class. The mIOU for intensity only from the third experiment decreased only about four percentage points to 68 compared to 72 for 3D coordinates with colour information from the first experiment. This shows that points with x, y, z, and intensity values without colour information (LiDAR-only data) can perform almost as well as 3D coordinates with colour information. In future work, we will analyse the impact of the stacking ensemble technique on our two models, namely the model trained on Semantic3D with RGB without intensity and the model trained on Semantic3D without RGB values but with intensity values.

## Figures and Tables

**Table 1:** A description of the Semantic3D (reduced-8) dataset classes.

Class	Description
Man-made	Man-made terrain, which is mostly pavement.
Natural	Natural terrain, which is mostly grass.
High vegetation	High vegetation, which is trees and large bushes.
Low vegetation	Low vegetation, which is flowers or small bushes that are smaller than 2 m of height.
Building	Houses, city halls, churches, stations, tenements, and so on.
Hardscape	A clutter class with garden walls, fountains, and banks.
Scanning arts	Artefacts caused by dynamically moving objects during static scan acquisition (this class may overlap with other classes, such as cars, if the cars are moving).
Cars	Cars and trucks.

**Table 2:** Comparative evaluation of the performance of different deep learning-based strategies on the SemanticKITTI dataset using the mean intersection over union (mIOU) metric. Larger values imply better performance.

Work	Description	Input	Type	mIOU
PointNet [6]	Takes into account the irregularity of point clouds; transformation-invariant	3D points	Raw data	14.60
PointNet++ [30]	An extension of PointNet where each local location is passed independently to a symmetric function	3D points	Raw data	20.10
RangeNet++ [39]	Rasterises the data points as range images	2.5D data	Range images	52.20
RandLANet [7]	A subset of data points is randomly selected to be able to run in real time	3D points	Raw data	**53.90**

**Table 3:** Comparative evaluation of the performances of different deep learning-based strategies on the Modelnet40 dataset [38] using the mean intersection over union (mIOU) metric. Larger values imply better performance.

Work	Description	Input	Type	mIOU
Volumetric [27]	Convert data points to voxels	Voxels	Transformed 3D points	83.00
MVCNN [28]	Obtains 12 views of an object	2D images	Transformed 3D points	90.10
3dmfv [29]	Converts data into Fisher vectors	Grid	Transformed 3D points	**91.40**

**Table 4:** Summary of the desired features of the solutions proposed in the literature compared to our proposed approach. A large scale of points refers to hundreds of thousands of points or more.

Solution	Runs in Real Time	Raw 3D Data as Input	LiDAR Only	Scale of Points	Sampling Approach
Volumetric [27]			✓	Unknown	N/A
MVCNN [28]	✓		✓	Unknown	N/A
PointNet [6]		✓	✓	Small	FPS
3dmfv [29]			✓	Small	N/A
PointNet++ [30]		✓	✓	Small	FPS
RangeNet++ [39]	✓		✓	**Large**	N/A
RandLANet [7]	✓	✓	✓	**Large**	Random
**Ours**	✓	✓	✓	**Large**	**Systematic**

**Table 5:** Benchmarking our systematic sampling algorithm with other 3D point selection algorithms, as reported by RandLA-Net. Smaller values imply better performance.

Algorithm	Complexity
Policy gradient-based sampling [49]	does not converge for large data
Farthest point sampling [50]	$O(N^{2})$
Inverse density importance sampling [51]	O(N)
Random sampling [7]	O(1)
**Ours**	O(NlogN)

**Table 6:** Benchmarking our network with the state-of-the-art RandLA-Net on the Semantic3D (reduced-8) dataset with x, y, z, and RGB values and no intensity. Larger values imply better performance.

	XYZ + RGB	
	**RandLA-Net**	**Ours**
mIOU (%)	69.31	**72.23**
Overall accuracy (%)	89.50	**91.30**
Man-made (IOU%)	94.09	**94.74**
Natural (IOU%)	81.87	**85.72**
High veg. (IOU%)	85.39	**89.52**
Low veg. (IOU%)	37.09	**39.49**
Building (IOU%)	82.18	**85.77**
Hardscape (IOU%)	25.71	**28.85**
Scanning arts (IOU%)	61.36	**62.95**
Cars (IOU%)	86.80	**90.77**

**Table 7:** Benchmarking our network with the state-of-the-art RandLA-Net on the Semantic3D (reduced-8) dataset, omitting RBG features. Larger values imply better performance.

	XYZ Only	
	**RandLA-Net**	**Ours**
mIOU (%)	53.79	**62.25**
Overall accuracy (%)	81.00	**85.60**
Man-made (IOU%)	77.39	**81.74**
Natural (IOU%)	31.44	**41.07**
High veg. (IOU%)	70.54	**83.33**
Low veg. (IOU%)	23.53	**34.38**
Building (IOU%)	**89.95**	89.92
Hardscape (IOU%)	17.36	**31.80**
Scanning arts (IOU%)	55.25	**59.66**
Cars (IOU%)	64.91	**76.09**

**Table 8:** Benchmarking our network with the state-of-the-art RandLA-Net on the Semantic3D (reduced-8) dataset, omitting RBG features but adding intensity information. Larger values imply better performance.

	XYZ + Intensity	
	**RandLA-Net**	**Ours**
mIOU (%)	47.01	**68.15**
Overall accuracy (%)	75.80	**89.23**
Man-made (IOU%)	76.22	**91.99**
Natural (IOU%)	17.51	**76.16**
High veg. (IOU%)	47.28	**84.06**
Low veg. (IOU%)	13.72	**38.63**
Building (IOU%)	**87.12**	85.18
Hardscape (IOU%)	17.85	**24.50**
Scanning arts (IOU%)	54.22	**62.89**
Cars (IOU%)	62.17	**81.79**

**Table 9:** Benchmarking our network with the state-of-the-art RandLA-Net on the Semantic3D (reduced-8) dataset with RBG features and intensity information. Larger values imply better performance.

	XYZ + Intensity + RGB	
	**RandLA-Net**	**Ours**
mIOU (%)	61.07	**67.93**
Overall accuracy (%)	85.20	**89.07**
Man-made (IOU%)	**93.14**	92.10
Natural (IOU%)	**82.72**	77.30
High veg. (IOU%)	64.89	**74.85**
Low veg. (IOU%)	23.32	**37.65**
Building (IOU%)	78.22	**91.46**
Hardscape (IOU%)	21.31	**26.93**
Scanning arts (IOU%)	53.71	**59.17**
Cars (IOU%)	71.26	**83.95**

**Table 10:** Benchmarking our network with the state-of-the-art RandLA-Net on the Semantic3D (reduced-8) dataset with RBG features as the baseline for the four experiments we conducted. Larger values imply better performance.

	RandLA-Net	x, y, z + RGB	x, y, z Only	x, y, z + Intensity	x, y, z + RGB + Intensity
mIOU (%)	69.31	**72.23**	62.25	68.15	67.93
Overall acc. (%)	89.50	**91.30**	85.60	89.23	89.07
Man-made (IOU%)	94.09	**94.74**	81.74	91.99	92.10
Natural (IOU%)	81.87	**85.72**	41.07	76.16	77.30
High veg. (IOU%)	85.39	**89.52**	83.33	84.06	74.85
Low veg. (IOU%)	37.09	**39.49**	34.38	38.63	37.65
Building (IOU%)	82.18	85.77	89.92	85.18	**91.46**
Hardscape (IOU%)	25.71	28.85	**31.80**	24.50	26.93
Scanning arts (IOU%)	61.36	**62.95**	59.66	62.89	59.17
Cars (IOU%)	86.80	**90.77**	76.09	81.79	83.95

**Table 11:** A description of the evaluation of the inference of SyS3DS on a GeForce RTX 3090 Nvidia GPU with 24 GB of memory on the Semantic3D (reduced-8) dataset with RBG features. The Semantic3D data with RGB features are the most complete data.

Total Frames per Point Cloud	Frames per Second	Seconds per Frame
4071 frames	24 frames per second	0.042

## Data Availability

Data are contained within the article.

## Appendix A

We have provided a video to show the qualitative results of our work on the Semantic3D dataset with x, y, z, and RGB values, which can be viewed at https://youtu.be/tqlkj-banys (accessed on 22 January 2024).
